# Impact of Brazil’s Bolsa Família Programme on cardiovascular and all-cause mortality: a natural experiment study using the 100 Million Brazilian Cohort

**DOI:** 10.1093/ije/dyac188

**Published:** 2022-09-28

**Authors:** Julia M Pescarini, Desmond Campbell, Leila D Amorim, Ila R Falcão, Andrêa J F Ferreira, Mirjam Allik, Richard J Shaw, Deborah C Malta, M Sanni Ali, Liam Smeeth, Mauricio L Barreto, Alastair Leyland, Peter Craig, Estela M L Aquino, Srinivasa Vittal Katikireddi

**Affiliations:** Centre for Data and Knowledge Integration for Health (CIDACS), Oswaldo Cruz Foundation, Salvador, Brazil; Departments of Infectious Disease Epidemiology (JMP) and Epidemiology and Population Health (LS), Faculty of Epidemiology and Population Health, London School of Hygiene & Tropical Medicine, London, UK; MRC/CSO Social & Public Health Sciences Unit, University of Glasgow, Glasgow, UK; Departamento de Estatística, Instituto de Matemática e Estatística, Universidade Federal da Bahia, Salvador, Brazil; Centre for Data and Knowledge Integration for Health (CIDACS), Oswaldo Cruz Foundation, Salvador, Brazil; Centre for Data and Knowledge Integration for Health (CIDACS), Oswaldo Cruz Foundation, Salvador, Brazil; MRC/CSO Social & Public Health Sciences Unit, University of Glasgow, Glasgow, UK; MRC/CSO Social & Public Health Sciences Unit, University of Glasgow, Glasgow, UK; Departamento materno infantil e saude pública, Universidade Federal de Minas gerais (UFMG), Belo Horizonte, Brazil; Departments of Infectious Disease Epidemiology (JMP) and Epidemiology and Population Health (LS), Faculty of Epidemiology and Population Health, London School of Hygiene & Tropical Medicine, London, UK; Departments of Infectious Disease Epidemiology (JMP) and Epidemiology and Population Health (LS), Faculty of Epidemiology and Population Health, London School of Hygiene & Tropical Medicine, London, UK; Health Data Research (HDR), London, UK; Centre for Data and Knowledge Integration for Health (CIDACS), Oswaldo Cruz Foundation, Salvador, Brazil; Instituto de Saúde Coletiva, Universidade Federal da Bahia, Salvador, Brazil; MRC/CSO Social & Public Health Sciences Unit, University of Glasgow, Glasgow, UK; MRC/CSO Social & Public Health Sciences Unit, University of Glasgow, Glasgow, UK; Instituto de Saúde Coletiva, Universidade Federal da Bahia, Salvador, Brazil; MRC/CSO Social & Public Health Sciences Unit, University of Glasgow, Glasgow, UK

**Keywords:** Cardiovascular disease, mortality, conditional cash transfer, Bolsa Família Programme, Family Health Strategy

## Abstract

**Background:**

Cardiovascular disease (CVD) has a disproportionate effect on mortality among the poorest people. We assessed the impact on CVD and all-cause mortality of the world's largest conditional cash transfer, Brazil’s Bolsa Família Programme (BFP).

**Methods:**

We linked administrative data from the 100 Million Brazilian Cohort with BFP receipt and national mortality data. We followed individuals who applied for BFP between 1 January 2011 and 31 December 2015, until 31 December 2015. We used marginal structural models to estimate the effect of BFP on all-age and premature (30–69 years) CVD and all-cause mortality. We conducted stratified analyses by levels of material deprivation and access to healthcare. We checked the robustness of our findings by restricting the analysis to municipalities with better mortality data and by using alternative statistical methods.

**Results:**

We studied 17 981 582 individuals, of whom 4 855 324 were aged 30–69 years. Three-quarters (76.2%) received BFP, with a mean follow-up post-award of 2.6 years. We detected 106 807 deaths by all causes, of which 60 893 were premature; and 23 389 CVD deaths, of which 15 292 were premature. BFP was associated with reductions in premature all-cause mortality [hazard ratio (HR) = 0.96, 95% CI = 0.94–0.98], premature CVD (HR = 0.96, 95% CI = 0.92–1.00) and all-age CVD (HR = 0.96, 95% CI = 0.93–1.00) but not all-age all-cause mortality (HR = 1.00, 95% CI = 0.98–1.02). In stratified and robustness analyses, BFP was consistently associated with mortality reductions for individuals living in the two most deprived quintiles.

**Conclusions:**

BFP appears to have a small to null effect on premature CVD and all-cause mortality in the short term; the long-term impact remains unknown.

Key Messages

- This is the first study to estimate the effect of a conditional cash transfer programme on cardiovascular and all-cause premature and all-ages mortality in a low- or middle-income country using individual-level data.
- The Bolsa Família Programme (BFP) was weakly and inconsistently associated with short-term cardiovascular and all-cause mortality in the general population.
- Associations between BFP and lower cardiovascular and all-cause mortality in more deprived municipalities persisted after robustness checks and should be better investigated.
- Longer follow-up, more consistent death registration in more deprived municipalities and further information on unmeasured confounding are needed to better estimate the full effects of BFP on mortality in Brazil.

## Introduction

Conditional cash transfer programmes (CCTs) are social policies that supplement incomes but also require beneficiaries to comply with conditions, such as participation in education or preventive healthcare.^1^ CCTs can tackle the structural and intermediate determinants of health and promote health equity by several mechanisms,^2^^,^^3^ such as by reducing poverty^4^^,^^5^ and improving diet and education take-up,^6^ and by contributing to women's empowerment and improved psychosocial circumstances (e.g. reduced indebtedness).^7^

In the past two decades, high rates of economic growth and the strengthening of social safety nets, such as CCTs, have played a substantial role in reducing economic and health disparities in low- and middle-income countries (LMICs).^2^ The Brazilian Bolsa Família Program (BFP) was implemented in 2004^8^ and is the largest and one of the oldest CCTs in the world.^6^^,^^9^ BFP had been delivered to >14.6 million families as of October 2021 and has had widespread uptake among all Brazilian poor households since 2010.^10^ Although BFP conditionalities focus on pregnant and breastfeeding women, children and adolescents, BFP cash benefits and conditionalities affect the entire household, as the programme has been associated with increased working hours, better jobs and higher familial income.^11–13^

Cardiovascular disease (CVD), the leading cause of death worldwide, is largely attributable to biological risk factors, such as high systolic blood pressure and high fasting plasma glucose, being overweight or obese and having an inadequate diet, that are disproportionally concentrated amongst poor individuals and those living in LMICs.^14^^,^^15^ In addition to behavioural factors and co-morbidities, premature mortality (i.e. death among persons aged 30–69 years)^16^ from CVD and all causes are strongly and consistently associated with low socio-economic status.^14^^,^^17^ Premature mortality is one of the Sustainable Developmental Goals indicators for monitoring effective policies for disease prevention and control.^18^^,^^19^ Therefore, policies aiming to reduce inequalities in CVD risk factors have great potential to reduce related deaths among the most disadvantaged populations. Nevertheless, there is limited evidence of the impact of CCTs on modifiable risk factors for CVD (e.g. tobacco and alcohol use and physical exercise) or on CVD mortality.^20^^,^^21^

In common with other CCTs, BFP may have ancillary benefits to adult health,^22^ such as reducing CVD risk through short-term (e.g. poverty reduction and improved nutritional status), short- to medium-term (e.g. use of preventive health services) and long-term (e.g. improved access to education and upward social mobility) mechanisms.^23^ Nevertheless, reductions in short-term CVD mortality due to CCTs are expected to occur mainly through greater knowledge about health and nutrition and increased access to healthcare services by beneficiary families,^22^ e.g. leading to the detection and treatment of adults in the family with severe CVD. We used data from the 100 Million Brazilian Cohort linked with mortality data in Brazil, a middle-income country,^24^ to estimate the short-term effect of BFP on premature and all-age cardiovascular mortality. Given the high contribution of CVD to all causes of death in Brazil, we further estimated the broader effects of BFP on all-cause mortality.^23^

## Methods

We followed a pre-specified protocol for this evaluation.^23^ Deviations from the original protocol are explained in the Supplementary material (available as Supplementary data at *IJE* online). The study is reported according to the reporting of studies conducted using the Template for Intervention Description and Replication (TIDieR) checklist^25^ (Supplementary Table S1, available as Supplementary data at *IJE* online).

### Intervention

Since BFP implementation, eligibility for BFP and income benefits varied to adjust for inflation.^23^ To be eligible for BFP, families must register with Brazil's National Registry for Social Programs ‘Cadastro Único’ (CadÚnico) and be extremely poor [monthly per-capita household income of ≤Brazilian Reais (BRL) 77 or USD 19 in 2015] or poor (monthly per-capita household income ≤BRL 154 or USD 39 in 2015).^26^ In 2015, families with income below the extreme poverty cut-off received a fixed benefit of BRL 77 (USD 19) plus a variable benefit of BRL 35–42 (USD 9–11) per child or adolescent, or pregnant or breastfeeding woman in the household. Families with income above the extreme poverty but below the poverty cut-off received only the variable benefit.^27^ Since 2011, extra benefits have been provided to families to ensure that their income is at least as high as the extreme poverty threshold after receiving BFP.^27^ Further details about the intervention, such as changes in the eligibility and amount over time, as well as details about BFP implementation, are included in the TIDieR-PHP^25^ reporting template (Supplementary Table S1, available as Supplementary data at *IJE* online).

### Study design and data sources

The 100 Million Brazilian Cohort^28^ is a dynamic cohort built from the registration records of individuals applying for benefits covered by the Unified Registry for Social Programs (CadÚnico) between 1 January 2001 and 31 December 2015, linked with BFP receipt and nationwide mortality records from the Mortality Information System.^29^^,^^30^ CadÚnico is used for the administration of all federal social assistance benefits within Brazil. It includes ∼55% of the total Brazilian population and has high coverage among low-income groups.^28^

From the cohort baseline (i.e. first registration in CadÚnico), we extracted socio-economic and demographic information at the individual (i.e. age, gender, race/ethnicity and education) and household level (i.e. region of residence, urban/rural residence, house construction material, electricity, water supply, sewage, garbage collection, household density and monthly per-capita income).^28^ For individuals aged <18 years, we assigned information on education as that of the household member with the highest level of education.

From the BFP database, we extracted information on the first and last date on which each household member received the benefit. We defined beneficiaries as individuals who were paid the benefit (usually a woman) or were members of a payee’s household. As BFP eligibility criteria (i.e. monthly per-capita income) changed over time, we standardized household monthly per-capita income using a correction factor that adjusted the income in line with the eligibility thresholds for the year of BFP application.^23^

From the Mortality Information System Database, we extracted information on the dates and causes of death. Deaths within Brazil are subject to certification by medical professionals, with cause of death coded according to International Classification of Diseases version 10 (ICD-10).^31^ Reporting rates in the Mortality Information System Database are considered high overall,^32^ reaching ≥90% completeness in 93% of municipalities, though there is some variation between municipalities, with lower levels of reporting in poorer municipalities, especially in the North.

We also extracted municipality-level information on (i) material deprivation,^17^ (ii) under-reporting of mortality and (iii) Family Health Strategy (FHS) coverage. Material deprivation was measured by population weighted quintiles of the Brazilian Deprivation Index (IBP)^33^ for 2010, which combines the proportion of households in a given area with per-capita income ≤1/2 minimum wage, proportion of those aged ≥7 years who are illiterate and the proportion of people with inadequate housing. Quintiles of IBP are used as they provide good variation but with small risk of misclassifying a municipality. Estimates of under-reporting for premature and all-age deaths, stratified by age and gender, were extracted for 2010.^34^ Coverage of the FHS,^35^ a community-based primary healthcare programme aimed to expand its access and which has been previously associated with lower all-cause and CVD mortality in Brazil,^36^ was available for 2015 (i.e. midpoint year of the study period).

### Data linkage

The linkage between the 100 Million Brazilian Cohort (2001–2015) and BFP data (2004–2015) was based on a single identifier (the National Identification Number or NIS). The linkage between the cohort baseline and mortality data (2001–2015) was performed in two steps using the CIDACS-RL tool.^37^ First, death records were linked based on exact matching, then unmatched records were linked using a similarity score. For both stages the matching was based on five identifiers (name, gender, year of birth, name of the mother and municipality of residency). The data set was constructed by the Centre for Data and Knowledge Integration for Health from Oswaldo Cruz Foundation,^30^ de-identified and provided to the researchers for use in a safe haven without access to the internet. Details on linkage sensitivity and specificity are in the Supplementary material (available as Supplementary data at *IJE* online).

### Study population

The study population consisted of individuals who registered with the 100 Million Brazilian Cohort from 1 January 2011 to 31 December 2015 as monthly per-capita income was only available from 2011 onwards. We excluded (i) individuals who apparently died before applying to the cohort, as the anomalous dates could reflect linkage errors and^17^ (ii) those who were >100 years of age at registration most likely have missing or unmatched death certificates. In addition, we excluded (iii) individuals with standardized monthly per-capita income of >BRL 300 (USD 75), so our study population was restricted to individuals who were more likely to be eligible for BFP; and (iv) individuals of Asian or Indigenous ancestry who together represented only 1.5% of the eligible population and whom we judged should not be grouped with other race/ethnicities because of their distinct characteristics. Finally, we excluded (v) individuals who applied on the last day of the follow-up (i.e. 31 December 2018) or died on the same day as they applied.

Our main analysis is related to individuals aged 30–69 years, reflecting premature mortality,^38^ but we also repeated the analysis for all individuals (aged zero up to 100 years).

### Primary and secondary outcomes

Our primary outcome was CVD mortality (ICD-10 I00–I99). Secondary CVD end points included the two main CVD subtypes, namely ischaemic heart disease (I20–I25) and cerebrovascular disease (I60–I69), and all-cause mortality.

For the primary analysis (premature mortality), the number of person-years at risk (pyr) that each individual contributed to the analysis started at registration or upon reaching 30 years of age and ended at the earliest of: (i) death, (ii) 31 December 2015 or (iii) when the individual reached 70 years of age. Individuals who applied to the 100 Million Brazilian Cohort before the age of 30 years and started receiving BFP before that date were considered as exposed to BFP from the start of their risk period. For the analysis of individuals from all ages, pyr of individuals aged 0–100 years started at registration and ended at death or on 31 December 2015, whichever came first.

### Analysis

We calculated directly age-standardized rates of CVD mortality amongst recipients and non-recipients using the Brazilian 2015 official population projection as the standard^39^ and estimated the 95% CIs according to Breslow and Day.^40^

We used a marginal structural model (MSM) using inverse probability of treatment weighting (IPTW) aiming to estimate the causal effect of BFP on mortality as BFP receipt is an exposure that varies over time.^41^ The remaining covariates were considered fixed in time. We classified individuals as unexposed prior to BFP receipt and exposed from the date of the first receipt, so we could compare exposed and non-exposed individuals with similar probabilities of being eligible at a given point in time. First, we estimated the probability of receiving BFP (hereafter referred to as the propensity score—PS) over time using logistic regression given their baseline socio-economic and demographic characteristics (i.e. gender, age, race/ethnicity, education, urban/rural area of residency, household building material, sanitation, household crowding, region of residency and year of application). Time of follow-up was also included as a covariate to estimate the PS using a smooth function based on cubic splines (with knots at the 5th, 25th, 50th, 75th and 95th percentiles).^42^ We calculated this probability up to the first month in which each individual started receiving the BFP and assumed that, once they start receiving the benefit, the probability was 1.

We used the propensity scores to derive IPTWs. Individuals’ attributed weights in each month *t* were equal to 1-PS when not receiving BFP and equal to the PS when receiving BFP. To estimate individuals’ probability of treatment up to each month since the start of the follow-up, the final assigned weight was calculated by multiplying the weights cumulative over time—i.e. we multiplied the weight in the month *t* by the 1 in month *t–1*. Finally, the effect of BFP on mortality outcomes was estimated through the hazard ratio (HR) using pooled logistic regression with time-varying weights and cluster robust standard errors to account for individuals contributing to the analysis at multiple time points. We first obtained the effect of BFP overall and then fitted stratified models for individuals living in richer and poorer areas, measured using the IBP,^33^ and those living in municipalities with different coverages of the FHS.^35^ As region of residency is also a proxy of socio-economic status; in models stratified for IBP levels, we removed it from the PS.

We considered that once individuals start receiving BFP, they are exposed for the remaining study period because (i) in our cohort, only 0.33% of people starting to receive BFP recipients stopped before the end of the follow-up;^17^ (ii) once eligible to receive BFP, recipients are required to update their registry once every 2 years or every time there is any change in address or income, and if their income increases above the eligibility threshold they continue to receive benefits for up to 2 years; and (iii) ancillary benefits (i.e. increased access to the Brazilian Universal Health care system and education) are expected to continue after the end of the cash benefit (Supplementary Table S1, available as Supplementary data at *IJE* online).

We calculated the e-values to assess the sensitivity of our results to potential unmeasured confounding.^43^^,^^44^ To test the robustness of our findings, we estimated the effect of BFP on mortality by (i) using time-varying Cox regression models adjusted for socio-economic and demographic covariates, (ii) weighting individuals by the inverse probability of receiving the treatment (IPTW), (iii) excluding individuals who received BFP on the same day as they applied to CadÚnico and (iv) implementing risk set matching that matches benefit recipients to non-recipient controls based on time and propensity score (further details in Supplementary File, Section 2, available as Supplementary data at *IJE* online). In addition, we used MSM to explore the potential for differential effects of BFP by conducting stratified analyses by age groups. The estimated effect of BFP on mortality could relate to poorer data quality and under-reporting of mortality among beneficiaries, which could introduce selection bias during linkage. To investigate this, we estimated the effect of BFP for individuals living in municipalities with <0.5% probability of death being underreported^34^ (i.e. ∼38% of studied individuals).

Analyses and visualizations were performed in STATA 16 and R Software version 3.6.0.

## Results

**Figure 1:**
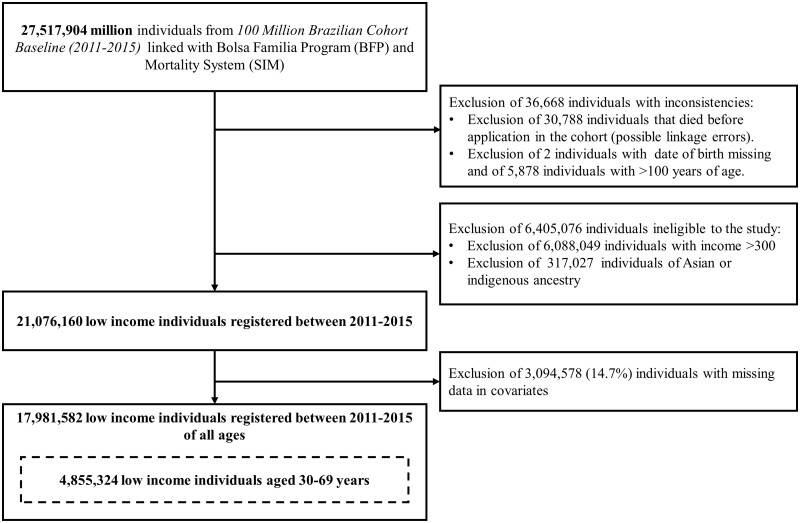
Flowchart of the study population

Our analytical cohort consisted of 17 981 582 low-income individuals, of whom 4 855 324 were aged 30–69 years for at least 1 day during follow-up (Figure 1). Among individuals aged 30–69 years, 65.5% (*n* = 3 177 839) received BFP at some time during the study period. Among individuals from all age groups, 76.2% (*n* = 13 705 334) received BFP benefits. Individuals were followed for ≤5 years (mean = 2.6, SD = 1.3), with similar follow-up among non-beneficiaries (mean = 2.6 and SD = 1.4 for both groups). BFP beneficiaries and non-beneficiaries differed systematically in all baseline demographic and socio-economic characteristics except gender (Table 1).

**Table 1:** Description of study population with monthly per-capita income <300 Brazilian Reais according to individuals who received Bolsa Família Programme at any point in time

	30 to 69 years (N = 4 855 324)					0–100 years (N = 17 981 582)				
Characteristics^a^	Non-BFP (*N* = 1 677 485)		BFP (*N* = 3 177 839)			Non-BFP (*N* = 4 276 248)		BFP (*N* = 13 705 334)		
	*N*	%	*N*	%	SMD	*N*	%	*N*	%	SMD
**Socio-demographic characteristics**										
Gender										
1: Men	753 821	44.9	1 577 715	49.6	0.094	2 011 476	47.0	6 686 079	48.8	0.035
2: Woman	923 664	55.1	1 600 124	50.4		2 264 772	53.0	7 019 255	51.2	
Age at entry year (years)										
0–9	–	–	–	–		1 537 376	36.0	7 938 768	57.9	0.503
10–19	–	–	–	–		564 209	13.2	1 500 664	10.9	
20–29	–	–	–	–		604 199	14.1	1 566 730	11.4	
30–39	788 781	47.0	1 885 654	59.3	0.295	610 547	14.3	1 363 101	9.9	
40–49	384 817	22.9	682 249	21.5		384 817	9.0	682 249	5.0	
50–59	316 806	18.9	437 889	13.8		316 806	7.4	437 889	3.2	
60–69	187 081	11.2	172 047	5.4		185 233	4.3	173 895	1.3	
≥70	–	–	–	–		73 061	1.7	42 038	0.3	
Race/ethnicity										
1: White	673 562	40.2	1 024 016	32.2	0.166	1 739 260	40.7	4 359 075	31.8	0.187
2: Black	119 742	7.1	268 516	8.4		226 514	5.3	756 100	5.5	
4: Mixed/brown	884 181	52.7	1 885 307	59.3		2 310 474	54	8 590 159	62.7	
Education										
0: Never went to school	122 214	7.3	268 332	8.4	0.104	453 586	10.6	1 953 519	14.3	0.273
1: Primary school or less (≤5 years of education)	574 848	34.3	1 140 992	35.9		965 051	22.6	3 369 267	24.6	
2: Junior high school (≤9 years of education)	308 298	18.4	648 500	20.4		828 528	19.4	3 618 895	26.4	
3: High school or more	672 125	40.1	1 120 015	35.2		2 029 083	47.5	4 763 653	34.8	
**Household characteristics**										
Region										
North	122 200	7.3	344 912	10.9	0.202	377 069	8.8	1 752 349	12.8	0.263
Northeast	425 988	25.4	932 245	29.3		1 078 001	25.2	4 496 826	32.8	
Southeast	739 346	44.1	1 365 194	43.0		1 777 195	41.6	5 183 838	37.8	
South	219 598	13.1	294 465	9.3		592 845	13.9	1 261 178	9.2	
Central-west	170 353	10.2	241 023	7.6		451 138	10.5	1 011 143	7.4	
Area of residence										
1: Urban	1 490 072	88.8	2 705 036	85.1	0.110	3 792 111	88.7	1 151 6822	84.0	0.136
2: Rural	187 413	11.2	472 803	14.9		484 137	11.3	2 188 512	16.0	
Household material										
1: Masonry/brick	1 512 796	90.2	2 753 774	86.7	0.115	3 820 708	89.3	1 153 6906	84.2	0.171
2: Coated or uncoated Taipa, wood, others	50 454	3.0	149 023	4.7		117 671	2.8	778 256	5.7	
4: Wood	114 235	6.8	275 042	8.7		337 869	7.9	1 390 172	10.1	
Water supply										
1: General network distribution	1 434 886	85.5	2 535 683	79.8	0.152	3 636 402	85.0	10 666 297	77.8	0.186
2: Well, spring or other	242 599	14.5	642 156	20.2		639 846	15.0	3 039 037	22.2	
Sewage disposal										
1: Public network	1 023 448	61.0	1 741 815	54.8	0.137	2 527 127	59.1	6 914 015	50.4	0.188
2: Septic tank	230 175	13.7	448 559	14.1		607 759	14.2	2 028 978	14.8	
3: Rudimentary trench, open ditch, water or others	423 862	25.3	987 465	31.1		1 141 362	26.7	4 762 341	34.7	
Waste disposal/garbage collection										
1: Collected directly/indirectly	1 521 950	90.7	2 724 790	85.7	0.155	3 885 804	90.9	11 487 414	83.8	0.213
2: Burned, buried or outdoor	155 535	9.3	453 049	14.3		390 444	9.1	2 217 920	16.2	
Electricity										
1: Electric with meter	1 496 865	89.2	2 608 592	82.1	0.223	3 730 601	87.2	10 852 556	79.2	0.247
2: Electric with community meter	95 269	5.7	230 189	7.2		293 160	6.9	1 105 544	8.1	
3: Informal electric lights or no electricity	85 351	5.1	339 058	10.7		252 487	5.9	1 747 234	12.7	
Household crowding (tercile)										
1: ≤0.75 individuals per room	1 099 732	65.6	1 437 185	45.2	0.492	2 428 161	56.8	4 779 523	34.9	0.557
2: 0.76–1 individuals per room	414 965	24.7	918 666	28.9		1 234 841	28.9	4 023 979	29.4	
3: >1 individual per room	162 788	9.7	821 988	25.9		613 246	14.3	4 901 832	35.8	
Sanitation										
All four components adequate	936 265	55.8	1 501 801	47.3	0.220	2 309 424	54.0	5 850 168	42.7	0.284
Three adequate	473 010	28.2	917 198	28.9		1 250 480	29.2	4 149 664	30.3	
Two adequate	155 409	9.3	398 203	12.5		417 966	9.8	1 903 635	13.9	
One or none adequate	112 801	6.7	360 637	11.3		298 378	7.0	1 801 867	13.1	
Year of application										
2011	338 964	20.2	798 878	25.1	0.244	661 715	15.5	2 980 612	21.7	0.276
2012	496 617	29.6	993 040	31.2		1 078 825	25.2	3 808 703	27.8	
2013	221 735	13.2	546 656	17.2		674 983	15.8	2 671 607	19.5	
2014	365 763	21.8	522 933	16.5		1 039 431	24.3	2 487 230	18.1	
2015	254 406	15.2	316 332	10.0		821 294	19.2	1 757 182	12.8	
Per-capita income (BRL)										
0–50	590 974	13.8	6 428 385	46.9	<0.001	165 441	9.9	1 324 976	41.7	<0.001
50–150	812 395	19.0	6 015 398	43.9		313 201	18.7	1 454 021	45.8	
150–300	2 872 879	67.2	1 261 551	9.2		1 198 843	71.5	398 842	12.6	

SMD, standardized mean difference prior weighting or matching; BFP, Bolsa Família Programme; BRL, Brazilian Reais.

a Distribution excluding missing data.

Age-standardized CVD mortality was 170.4/100 000 pyr overall and higher among BFP beneficiaries (Rate = 178.8/100 000 pyr) than among non-beneficiaries (Rate = 164.5/100 000 pyr) (Table 2). The two main specific causes of CVD mortality, i.e. ischaemic heart disease (7471/23 389 or 31.9% of CVD deaths) and cerebrovascular disease mortality (6722/23 389 or 28.7%), were also higher among BFP recipients. Similarly, age-standardized all-cause mortality was higher among beneficiaries (Rate = 607.0/100 000 pyr) than among non-beneficiaries (Rate = 567.6/100 000 pyr). Mortality was generally higher among men than women, apart from cerebrovascular disease mortality, for which the rates were similar across genders (Table 2).

**Table 2:** All-cause, cardiovascular, ischaemic heart disease and cerebrovascular disease age-standardized mortality rates overall and by Bolsa Família Programme receipt, 2011–2015

	Overall (N = 17 981 582)			Non-BFP (N = 4 276 248)			BFP (N = 13 705 334)		
All age groups	Events	Person-years at risk	Age-standardized rate^a^^,^^b^ (95% CI)^c^	Events	Person-years at risk	Age-standardized rate^a^^,^^b^ (95% CI)^c^	Events	Person-years at risk	Age-standardized rate^a^^,^^b^ (95% CI)^c^
CVD mortality	23 389	47 869 832	170.4 (170.4–170.4)	12 195	15 144 018	164.5 (164.5–164.5)	11 194	32 731 881	178.8 (178.8–178.8)
Men	13 352	22 973 212	177.1 (177.1–177.1)	6644	6 974 914	177.3 (177.3–177.3)	6708	16 001 934	178.6 (178.6–178.6)
Women	10 037	24 896 620	162.3 (162.3–162.3)	5551	8 169 104	154.9 (154.9–154.9)	4486	16 729 947	174.4 (174.3–174.4)
Ischaemic heart disease mortality	7471	47 869 832	52.7 (52.7–52.7)	3858	15 144 018	50.7 (50.7–50.7)	3613	32 731 881	55.2 (55.1–55.2)
Men	4738	22 973 212	60.9 (60.9–60.9)	2347	6 974 914	61.6 (61.6–61.6)	2391	16 001 934	60.5 (60.5–60.5)
Women	2733	24 896 620	43.7 (43.7–43.7)	1511	8 169 104	41.5 (41.5–41.5)	1222	16 729 947	46.8 (46.8–46.8)
Cerebrovascular disease mortality	6722	47 869 832	52.6 (52.5–52.6)	3552	1 514 4018	50.0 (50.0–50.0)	3170	32 731 881	56.5 (56.5–56.5)
Men	3675	22 973 212	52.8 (52.8–52.8)	1862	6 974 914	51.8 (51.8–51.8)	1813	16 001 934	54.5 (54.5–54.5)
Women	3047	24 896 620	51.6 (51.6–51.6)	1690	8 169 104	48.4 (48.4–48.4)	1357	16 729 947	57.2 (57.2–57.2)
All-cause mortality	106 807	47 869 832	585.5 (585.5–585.5)	48 332	1 514 4018	567.6 (567.6–567.6)	58 475	32 731 881	607.0 (606.9–607.0)
Men	65 045	22 973 212	670.3 (670.3–670.3)	28 083	6 974 914	672.2 (672.2–672.2)	36 962	16 001 934	668.7 (668.6–668.7)
Women	41 762	24 896 620	507.7 (507.7–507.7)	20 249	8 169 104	487.7 (487.7–487.8)	21 513	16 729 947	539.9 (539.9–539.9)

BFP, Bolsa Família Programme; CVD, cardiovascular disease.

a Rates were calculated considering the treatment to vary over time (i.e. follow-up time for individuals exposed to BFP were calculated separately before the intervention and allocated to the non-BFP group and later allocated to the BFP group).

b The mortality rates were standardized by age (in 5-year age groups) using 2015 Brazilian population. Data available at http://tabnet.datasus.gov.br/cgi/deftohtm.exe?popsvs/cnv/popbr.def.

c The 95% CI estimates take into account the method described by Breslow and Day to calculate the standard error and assume that the numbers of events in each age group follow a Poisson distribution.^42^

**Figure 2:**
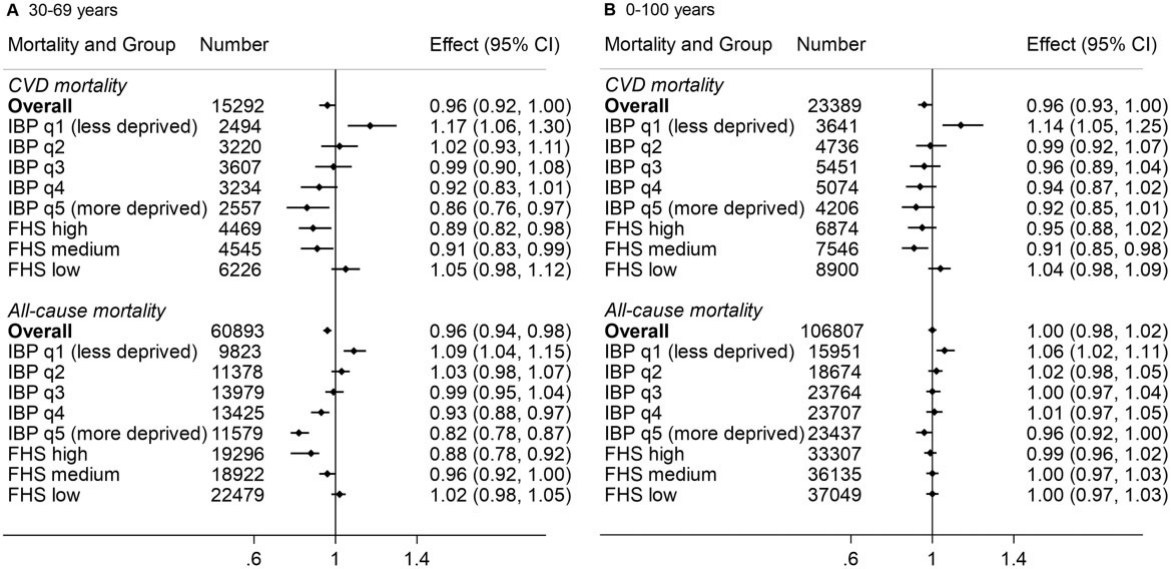
Estimation of the marginal structural model effect of Bolsa Família Programme receipt on cardiovascular and all-cause mortality for individuals living in municipalities with high-low material deprivation and high-low coverage of the Family Health Strategy IBP, Brazilian Deprivation Index (divided into quintiles); FHS, Family Health Strategy (divided into terciles); CVD, cardiovascular diseases; q1, quintile 1; q2, quintile 2; q3, quintile 3; q4, quintile 4; q5: quintile 5. For the 30–69 years age group, 709 premature all-cause mortality and 180 CVD premature mortality missing values for IBP levels and 196 premature all-cause mortality and 52 CVD premature mortality missing values for FHS coverage. For the 0–100 years age group, 1274 all-cause mortality and 280 CVD mortality missing values for IBP levels and 316 all-cause mortality and 69 CVD mortality missing values for FHS coverage. For the 30–69 years age group, FHS coverage is divided into terciles of high (≥83%), medium (44–83%) or low coverage (≤44%). For the 0–100 years age group, FHS coverage is divided into terciles of high (≥87%), medium (44–87%) or low coverage (≤44%).

BFP receipt was associated with 4% lower CVD (HR = 0.96, 95% CI = 0.92–1.00) and all-cause (HR = 0.96, 95% CI = 0.94–0.98) premature mortality (Figure 2). When stratified by municipality-level deprivation, BFP receipt was associated with a lower risk of CVD premature mortality in more deprived municipalities and those where FHS had high or medium coverage (≥44%). In contrast, BFP receipt was associated with higher mortality for individuals living in less deprived municipalities (HR = 1.17, 95% CI = 1.06–1.30). Similarly, lower all-cause premature mortality among BFP recipients was found for individuals living in the most deprived municipalities (fourth quintile: HR = 0.93, 95% CI = 0.88–0.97; fifth quintile: HR = 0.82, 95% CI = 0.78–0.87). BFP receipt was associated with higher mortality for individuals living in the least deprived municipalities (first quintile: HR = 1.09, 95% CI = 1.04–1.15).

Among individuals of all ages, BFP receipt was not associated with CVD mortality (HR = 0.96, 95% CI = 0.93–1.00) or all-cause mortality (HR = 1.00, 95% CI = 0.98–1.02) (Figure 2). Similar effects on all-age mortality were found after stratifying by municipal-level deprivation (i.e. reductions among more deprived and higher mortality among less deprived) and FHS coverage. E-values for the HRs ranged between 1.16 and 1.74 for premature mortality and 1 and 1.54 for all-ages mortality, showing the highest value (E-value = 1.74) for the more deprived municipalities (i.e. IBP quintile 5) for all-cause premature mortality.

Robustness checks using time-varying and IPTW Cox models, MSM excluding individuals who received benefit as soon as they applied to CadÚnico and risk set matching yielded inconsistent findings, suggesting a null or higher chance of mortality among BFP beneficiaries (Supplementary Table S4, available as Supplementary data at *IJE* online).

The analysis by age group showed generally similar results by age for CVD and all-cause premature mortality (Supplementary Table S5, available as Supplementary data at *IJE* online). However, for mortality among individuals of all ages, the effect estimates followed an inverted U-shaped curve with highest estimates being observed for individuals aged 50–69 years for both CVD and all-cause mortality (Supplementary Table S5, available as Supplementary data at *IJE* online). When restricting the analysis to individuals living in municipalities with less under-reporting of deaths (Supplementary Table S6, available as Supplementary data at *IJE* online), we found no consistent association between receiving BFP and lower risk of CVD or all-cause mortality, but the association between BFP receipt and lower CVD and all-cause premature mortality in more deprived municipalities persisted.

## Discussion

This is the first study to estimate the effect of a conditional cash transfer programme on cardiovascular and all-cause premature mortality in low- or middle-income countries using individual-level data. We analysed longitudinal data from >17 million low-income individuals followed for ≤5 years. We observed small to null effects of the Brazilian conditional cash transfer BFP on CVD and all-cause premature mortality, but larger and consistent mortality reductions in the areas with higher levels of deprivation.

In Brazil, although mortality from premature CVD is high, rates declined from 294.3 to 153.9 per 100 000 inhabitants from 1990 to 2017.^45^ This has been accompanied by a high prevalence of CVD risk factors, which are more prevalent among women receiving BFP (e.g. overweight, obesity, tobacco, hypertension and diabetes).^46^ Although BFP recipients have health disadvantages compared with the general Brazilian population before accounting for socio-economic status,^46^ there is evidence of a positive effect of BFP receipt on the overall expenditure on food (both healthy and ultra-processed and high-caloric food)^47^ and on the increased consumption and expenditure on healthy foods (i.e. fresh or minimally processed food).^48^^,^^49^

The consistent estimates for the effect of BFP receipt on lower CVD and all-cause premature mortality in more deprived municipalities in Brazil could be explained by higher homogeneity of individuals’ socio-economic characteristics, which could lead to lower unmeasured confounders. In addition, in the most vulnerable municipalities, the lower prices per calorie of natural products and cooking ingredients in the poorest and rural regions of Brazil could translate into a higher purchasing power.^49^^,^^50^ Conversely, income transfers in wealthier and larger municipalities, which have greater availability and consumption of ultra-processed and caloric food, may exacerbate being overweight and obesity, which are important risk factors for non-communicable diseases.^4^^,^^5^^,^^51^ In theory, these mechanisms could lead to overall null effects when looking at the effect of BFP receipt in a large and heterogeneous country like Brazil.

The null association between BFP and CVD premature mortality after accounting for under-reporting, especially in places with lower FHS coverage, may also indicate improved mortality registration among families that are enrolled in both BFP and FHS programmes. The BFP health conditionalities might encourage uptake of health checks and nutrition education and surveillance among all household members, as seen by increased tuberculosis and leprosy cure rates among BFP beneficiaries in Brazil.^52^^,^^53^ A previous study with the CCT Pregressa in Mexico found that adults from beneficiary families that participated in the programme for 3.5–5 years had a lower prevalence of obesity and hypertension compared with non-beneficiaries.^54^ Adults, especially women, are more likely to attend health services during conditionality check-ups and can benefit from contact with health professionals, being monitored or diagnosed for co-morbidities such as hypertension or obesity.^22^^,^^54^ The majority of BFP beneficiary families are monitored by the FHS, which provides an interdisciplinary healthcare team that contributes to improved access to the health system.^55^ Monitoring of health conditionalities could also help in improving access to the health system, contribute to the adoption of healthy eating and physical activity behaviours, promote early CVD diagnosis and increase access to antihypertensive medications of adults.^23^^,^^36^^,^^55^ The increased coverage of FHS in Brazil in the last decade, especially in smaller and poorer municipalities,^56^ has also been associated with improved follow-up of chronic conditions and infectious diseases, as well as the reduction in hospitalization rates across Brazil.^36^^,^^55^^,^^57^ Nevertheless, the effectiveness of social interventions on the socio-economic determinants of health and health outcomes are largely dependent on other factors, such as political will, macro-economic stability, household dynamics and community acceptance.^58^

Our study took advantage of a large sample size with rich socio-economic data of CadÚnico linked with nationwide mortality data to provide a unique opportunity to evaluate the short-term effect of BFP receipt on all-cause and CVD mortality among the poorest individuals of Brazil. However, our study also has limitations. First, we used secondary administrative data whose quality varies across regions. As poor data quality can increase information bias arising in the linkage process, we repeated our analysis restricted to municipalities with better mortality data and found a consistent association of BFP receipt on lower CVD and all-cause mortality among individuals living in the two most deprived municipality quintiles and in municipalities with higher coverage of FHS. Second, due to the impossibility of applying a fuzzy regression discontinuity design, we used the MSM developed by Robins,^41^ given that this is the most well-described causal method that can be used to deal with time-varying variables such as BFP receipt and survival. However, robustness checks using different statistical methods found inconsistent associations between receiving BFP and mortality. Although risk set matching might have accounted for municipal variations in BFP implementation or other unmeasured confounders, individuals who received BFP straight away (i.e. in the first few days after applying to CadÚnico) were unlikely to be matched due to their low propensity scores obtained in the Cox regression model, suggesting that such individuals were systematically different from those who received BFP later. Third, for a causal interpretation of the effect of BFP on mortality, MSM assumes no unmeasured confounding. In our analysis, we were able to control for many socio-demographic confounders, including age, but we were unable to control for the main behavioural risk factors for CVD premature mortality (i.e. excess bodyweight, unhealthy diet, tobacco, harmful use of alcohol, hypertension and physical inactivity)^17^ or access to secondary or tertiary care. However, by stratifying by municipal-level deprivation, we possibly have accounted for some variations in the tobacco and alcohol consumption,^59^^,^^60^ as well as in availability and access to hospital beds. In addition, controlling for behavioural risk factors, which we were unable to do, is not necessarily appropriate as they lie on the causal pathway between socio-economic factors and CVD. Although small, the e-values obtained in our estimates suggested a null effect of BFP in the presence of unmeasured confounders. However, interpreting e-values depends on the measured confounders that were accounted for in the analysis—in our case, we accounted for multiple socio-economic confounders. In addition, we must consider that e-values are less useful in the presence of multiple, possibly interacting unmeasured confounders, which could be the case for socio-economic and behavioural factors.^43^^,^^61^

Fourth, as income data from CadÚnico were poorly recorded before 2011, we were only able to study new applicants to BFP from 2011 onwards and to follow individuals for ≤5 years. Thus the data only allowed investigation of short-term effects of BFP. Longer follow-up might be needed to assess the full effect of BFP receipt on all-cause and CVD mortality.

Despite these limitations, our study adds to the limited body of evidence on the effects of CCTs on CVD mortality in LMICs. A previous analysis of the Progresa CCT scheme using municipal-level data on Mexican elders (≥65 years) found that receipt was associated with a 4% reduction in elderly all-cause mortality but not associated with CVD mortality in the same group.^20^ In contrast, another ecological study using a similar difference-in-difference approach but assessing the Mexican non-conditional pension scheme ‘70 y Más’ targeting elders found, on average, a 5% increase in all-cause mortality among recipients, driven by increases in deaths related to CVD in more economically disadvantaged communities.^21^

## Conclusions

Social protection has been associated with improved access to healthcare and health outcomes among individuals residing in LMICs^4^^,^^62^ and constitutes a viable policy option for poverty reduction.^63^ By following millions of Brazilians (nearly 34 million people as in 2015 in CadÚnico^23^) for ≤5 years, we found weak evidence that BFP receipt might lead to small reductions in premature and all-age CVD mortality in the short term across the entire country. Beneficial effects seem strongest for populations living in more deprived municipalities. Future research should seek to identify the longer-term effects of participation in CCTs on cardiovascular and all-cause adult mortality and, if present, the mechanisms by which those effects occur.

## Ethics approval

This study was performed under the international (Helsinki), Brazilian and UK research regulations. The 100 Million Cohort Study and this study were approved by ethics committees from Instituto Gonçalo Muniz—Oswaldo Cruz Foundation (1 612 302 in 2016 and 4 243 677 in 2020) and the University of Glasgow, Medical, Veterinary and Life Sciences College (200190001).

## Supplementary Material

dyac188_Supplementary_Data

## Data Availability

The data underlying this article will be shared on reasonable request to CIDACS Fiocruz and after ethical approval.
