# Supplementary material for: Impact of Brazil’s Bolsa Família Programme on cardiovascular and all-cause mortality: a natural experiment study using the 100 Million Brazilian Cohort
Source: Int J Epidemiol. 2022 Sep 28;51(6):1847–61. doi: 10.1093/ije/dyac188 (PMC9749722; doi:10.1093/ije/dyac188)
Supplement: dyac188_Supplementary_Data [file dyac188_supplementary_data.docx]

**SUPPLEMENTARY FILE**

# Data linkage

The linkage between the 100 Million Brazilian Cohort (2001-2015) and Bolsa Familia Programme (BFP) data (2004-2015) was based on a single identifier (the National Identification Number or NIS) present in both databases. The linkage between the cohort baseline and mortality data (2001-2015) was performed in two-steps using the Centro de Integração de Dados e Conhecimentos para a Saúde record linkage system (CIDACS-RL) ^1^. In the first step, entries were exactly matched on five identifiers (name, sex, year of birth, name of the mother and municipality of residency). In the second step, entries that were not linked in the first step were linked based on a similarity score (i.e., ranging from 0 to 1) using this five identifiers^1^. Linkage sensitivity and specificity was calculated by year as the number of registries of CadÚnico, for which mortality registries were submitted to linkage, increased over time due to the dynamicity of the baseline cohort. The best cut-off point presented a sensitivity that ranged from 99.2% in 2011 to 98.4% in 2015, and specificity from 97.8% in 2011 to 94.3% in 2015.

# Deviations from protocol of analysis and robustness checks

## Deviations from protocol of analysis

In the protocol for this paper, we proposed to apply fuzzy regression discontinuity design (FRDD) oin the assumption that the probability of receiving BFP would increase gradually with the income and there would be a discontinuity at the cut-off. To assess the applicability of FRDD, we first investigated the main assumptions of (i) lack of manipulation of the assignment variable to receive BFP, exposure groups were exchangeable around the cut-off, and (iii) the probability of having the outcome is continuous in the absence of the intervention.

These investigations indicated that applying FRDD was not feasible to study the effects of BFP on mortality in the 100M cohort, meaning that we had to depart from the original plans and the published protocol ^2^. The key limitations were:

1. Evidence of manipulation of the assignment variable to receive BFP and lack of exchangeability around the cut-off. By investigation the distribution on monthly per capita income, the assignment variable for BFP, there is some evidence of manipulation around the threshold of poverty (154 Brazilian Reais (BRL)) and high variation in the distribution raising some concern about the exchangeability of individuals around the cut-off point (See Figure S1).
2. The probability of having the outcome seems not to be continuous giving the assignment variable (i.e., income) in the absence of the intervention. By plotting the distribution of deaths over income suggested that noise in the mortality data (even with large bandwidth) for different income values (i.e., unclear pattern between income and mortality) would make difficult to attribute changes in mortality in the cut-off point to the treatment (See Figure S2).

## Robustness checks

To test the robustness of our findings, we estimated the effect of BFP on Cardiovascular disease (CVD) and all-cause mortality by i. using an extension of Cox regression models with a time-varying exposure adjusted for socioeconomic and demographic covariates, ii. by weighting individuals by the inverse probability of receiving the treatment (IPTW), and iii. by risk set matching. Results are presented in Tables S2-S4.

2.1 Time varying Cox regression

To estimate the effect using time varying Cox regression model, study individuals contributed first as untreated (Non-BFP), and later as treated (BFP) if they ever received the treatment. We estimated the effect using multiple adjusted cox regression and with cluster robust standard errors to account for individuals contributing to multiple observations (i.e., maximum of two).

2.2 Cox regression weighting by inverse probability of treatments weights (IPTW)

To estimate the effect using IPTW, we defined BFP recipients as those individuals who first received the benefit within 6 months of joining to the cohort. The follow up time of each individual started at the 6^th^ month, ie., the point at which they were allocated to either the treatment or the non-treatment groups. In addition, individuals that began receiving BFP after the 6th month were additionally censored when they receive BFP. We used logistic regression to estimate the probability of individuals receive BFP within 6 months given the same socioeconomic and demographic covariates used in the primary analysis (See Table S2 and Figure S3). The average treatment effect on the treated (ATT) weights were estimated by weighting non-BFP recipients as the PS/(1-PS) and BFP recipients as 1, so to estimate the causal effect assuming the distribution of covariates in the untreated group is the same as in the treated. To reduce sparse weights, we truncated the weights at the 99 percentile ^4^. We estimated the effect of BFP on CVD using Cox regression analysis with ATT weights as sample weights (Table S4).

To check if the IPTW was able to balance non-BFP and BFP groups on observed covariates, we compared standardised mean differences (SMD) between the two groups before and after weighting, using SMD<0.1 as a criterion for successful control for each confounders^3^ (See Table S3).

2.3 Risk set matching

We used risk set matching analysis to investigate the effect of Bolsa Familia Programme (BFP) on cardiovascular and all-cause mortality. We used datasets consisting of (i) subjects of all ages and (ii) subjects aged between 30 and 69, to investigate effects on overall mortality and premature mortality respectively.

*Propensity Score Generation*

For each dataset, in each stratum defined by region and year of entry into the study, a survival model was fitted to model the delay from registration until receipt of BFP that included age, sex, race, area of residence, household building material, sanitation and household crowding as regressors. The propensity score for receipt of BFP was calculated for each subject. The linear predictor of each fitted survival model was used to predict log hazard ratio for each subject of the corresponding region year of entry stratum. The predictions were standardised to have zero mean and a standard deviation of one to give the propensity scores for the individuals.

The fitted survival models for delay until receipt of BFP had adjusted Rsq ranging from 0.15 to 0.25. The propensity scores generated from these models were approximately Gaussian and showed no signs of having significant populations with outlier values.

*Sequential matching*

Sequential pair matching was then performed. Subjects were sorted in order of their delay until BFP receipt. Unpaired subjects with the shortest delay were matched against subjects with longer delays. Matched pairs and any unmatchable shortest delay subjects were then removed from the matching process. The matching process was repeated until all subjects had either been paired or been found to be unmatchable. The result was a set of matched pairs of subjects.

The matching process is a computationally costly exercise which scales as the square of the number of subjects. It was performed in batches of up to 3000 subjects, with the smallest batch size being 1600. The batches were taken from strata defined by region, year of entry, sex and crowding, so paired subjects were always exactly matched on these categorical variables. A caliper was set so that the age difference between matched subjects could not exceed 10 years. Further they were also matched on propensity score, with a caliper set on the maximum allowable propensity score difference. Due to the computational cost of the matching process, matching was only performed on a random draw of 10 million subjects from the all ages dataset.

For each of the propensity score model regressors, the Absolute Standardised Mean Difference (ASMD) was used to assess the degree of balance between the pair member with the shorter delay to BFP receipt and the pair member with the longer delay to BFP receipt. The matching process resulted in approximately 55% of subjects being paired. The balance between the two types of pair members appeared to be good as ASMD was below 0.022 for all propensity score predictors (caution is advised if ASMD>0.1).

*Survival Analysis for mortality outcomes*

The intended analyses could suffer immortal time bias as the survival to receipt of BFP will be right censored by the end of follow up or end at the occurrence of death event. Transformation of the dataset from a row per person format to a row per interval format (using R survival::tmerge), allows the dataset to encode time dependent covariates and for immortal time bias to be overcome. Each pair of matched subjects formed a natural experiment in which the person with the shortest delay to receipt of BFP was treated and the other pair member was untreated. For each pair member, the interval was set to begin at the minimum within pair delay until treatment, and to end at the minimum of the delay until death and the maximum within pair delay until treatment.

For all scenarios, the effect of BFP on mortality outcomes was estimated using Cox regression analysis, where time until death was modelled in terms of delay until receipt of BFP while taking into account the matching of subjects. An additional survival analysis was also performed in which the propensity score predictors not exactly matched in the pairing process were added to the regression as regressors (Table S4).

# Supplementary Tables

## Table S1. TIDieR-PHP report

| **TIDieR-PHP reporting item** | **Programme description** |
| --- | --- |
| 1 Brief name | Bolsa Familia Programme (BFP) |
| 2 Why | A conditional cash transfer programme, intended to alleviate poverty and reduce intergenerational poverty, improving education and health, by providing eligible families with a regular income supplement conditional on school attendance and use of pre-/post-natal and child health services. |
| 3 What - materials | Cash benefits are paid to women (usually) in qualifying households. To be eligible households must be registered in the Brazilian National Registry for Social Programmes (Cadastro Unico), have an income below a defined poverty (income) threshold, and/or meet certain qualifying conditions: pregnant women must access prenatal care; children aged 0-5 and breastfeeding women must undergo monitoring by health professionals; and school age children must attend school for at least 80% (check) of school days.  Families defined as being in extreme poverty (monthly income below R$77 in 2014) receive a fixed benefit of R$77 plus additional amounts for pregnant women, children and adolescents up to a maximum of seven (from 2011; formerly five). Families with a monthly income of R$77-154 receive only the supplements for pregnant women, children and adolescents, if any in the household, and do not receive the fixed benefit. A number of changes to income thresholds for eligibility occurred from to 2004 (implementation year) onwards. Indigenous and quilombola communities (old African settlements) are prioritize to receive the benefit. |
| 4 What and How | BFP was established and is funded by the federal government, under law No 10,836 in 2003.  Payments are preferentially made to women and are credited directly to beneficiaries’ electronic benefit cards. The cash benefit can be withdraw and is be widely accepted in food and grocery shops. The cash benefit stays in the beneficiaries card for 90 days but return to the government the beneficiary do not use it.  Families receiving payments are monitored by social assistants to check compliance with the conditions. Compliance with the education conditions is monitored by schools. They provide attendance information to municipal education authorities, who calculate attendance rates and forward them to the Federal Ministry of Education. Those whose income increases above the eligibility threshold continue to receive benefits for up to two years. |
| 5 Who provided | The federal government allocated Bolsa Família program quotas of beneficiaries to municipalities according to estimates of poverty at the municipality level. Municipalities collect data on households and forward the information to a national database for final eligibility decisions.  Overall program and policy management, including the management of the national database, eligibility determination, and data verification, are conducted centrally by the Ministry of Social Development. The state bank Caixa Econômica Federal administers payments. |
| 6 Where | BFP is a national programme. It was intended to consolidate and extend pre-existing regional and national cash transfer programmes related to education, nutrition and household fuel costs. |
| 7 When and How often | BFP payments start transferred in January 2004, with a number of changes to the amount of benefits paid in subsequent years. |
| 8.1 Planned variation | Conditional on registration in Cadastro Unico, the criteria for selection into BFP differ by municipality. Beneficiaries are selected at the national level, through the following procedure. Each municipality is assigned a quota for a maximum number of BFP recipient households based roughly on poverty maps. If the number of households in a particular municipality with income per capita below the threshold is lower than the municipality’s quota, then all such households are selected for BFP. If the number of households in a particular municipality with income per capita below the threshold exceeds the municipality’s quota, priority is assigned for selecting households into BFP roughly according to the following criteria: (1) lower household income per capita, and (2) more children aged 0–17 years in the household. Consequently, while eligibility criteria are similar across municipalities, two households below the income per capita threshold with very similar characteristics may have different recipient status due to being in municipalities with different quotas and due to differences in the prioritization of eligibility criteria across municipalities. In 2003 the BFP target was estimated in 11 million families. Nevertheless, in 2015 it was estimated that around 15 million families are benefited yearly. |
| 8.2 Unplanned variation | All households are free to register in the Cadastro Unico. However, municipality-level officials are responsible for organizing the registration process, such that there is substantial heterogeneity across municipalities in targeting for Cadastro Unico registration, as well as in registration methods. |
| 9.1 How well | Central administration of assessment and payments is intended to address drawbacks in earlier programs designed to reduce poverty, such as their lack of transparency in identifying beneficiaries, political manipulation, and potential problems in correctly identifying the poor that might exacerbate inequalities. The BFP is equipped with fraud prevention control mechanisms and the suspension of benefits may occur due to non-updating of registration information, current non-compliance with the eligibility criteria and non-compliance with conditionalities. |
| 9.2 How well - delivery | Take-up rate can be measured if there is any beneficiary family that do not use the money in the card in up to 90 days, which should return to the government.  Compliance with educational and health conditionalities can be measured for children. Unfortunately, these datasets are still not linked with the present 100 Million Brazilian Cohort. |

Table S2. Logistic regression to estimate the propensity score for receiving or not receiving BFP within 6 months after applying to the cohort among 30 to 69 years old, and all ages (0-100 years old).

| **Covariates** | **30-69 years** | **0-100 years** |
| --- | --- | --- |
| *Individual characteristics* |  |  |
| **Gender** |  |  |
| Men | 1 | 1 |
| Woman | 0.83 (0.83-0.83) | 1.02 (1.01-1.02) |
| **Age (at entry year)** | 0.98 (0.98-0.98) | 0.96 (0.96-0.96) |
| **Race/ethnicity** |  |  |
| White | 1 | 1 |
| Black | 1.26 (1.25-1.27) | 1.32 (1.31-1.33) |
| Mixed/brown | 1.15 (1.15-1.16) | 1.18 (1.17-1.18) |
| **Education** |  |  |
| Never went to school | 1 | 1 |
| Primary school or less (≤5 years of education) | 0.89 (0.89-0.9) | 0.97 (0.97-0.98) |
| Junior high school (≤9 years of education) | 0.84 (0.83-0.85) | 0.98 (0.98-0.99) |
| High school or more | 0.7 (0.69-0.7) | 0.65 (0.64-0.65) |
|  |  |  |
| *Socioeconomic characteristics* |  |  |
| **Region** |  |  |
| North | 1 | 1 |
| Northeast | 1.1 (1.09-1.1) | 1.26 (1.26-1.27) |
| Southeast | 1.26 (1.25-1.27) | 1.16 (1.16-1.17) |
| South | 0.85 (0.85-0.86) | 0.75 (0.75-0.75) |
| Central-west | 0.79 (0.79-0.8) | 0.73 (0.73-0.74) |
| **Area of residence** | |  |
| Urban | 1 | 1 |
| Rural | 0.94 (0.93-0.94) | 0.95 (0.94-0.95) |
| **Household material** | |  |
| Masonry / brick | 1 | 1 |
| Coated or Uncoated Taipa, wood, others | 0.94 (0.93-0.95) | 1.07 (1.07-1.08) |
| Wood | 1.14 (1.13-1.15) | 1.16 (1.16-1.17) |
| **Sanitation** |  |  |
| All 4 components adequate | 1 | 1 |
| Three adequate | 1.13 (1.13-1.14) | 1.17 (1.17-1.18) |
| Two adequate | 1.3 (1.29-1.3) | 1.39 (1.39-1.4) |
| One or none adequate | 1.46 (1.45-1.48) | 1.63 (1.62-1.64) |
| **Household crowding (tercile)** | | |
| <=0.75 individuals per room | 1 | 1 |
| 0.76-1 individuals per room | 1.73 (1.73-1.74) | 1.54 (1.54-1.55) |
| >1 individuals per room | 3.46 (3.44-3.48) | 3.18 (3.17-3.2) |
| **Year of application** | |  |
| 2011 | 1 | 1 |
| 2012 | 0.62 (0.61-0.62) | 0.7 (0.7-0.71) |
| 2013 | 0.91 (0.9-0.92) | 0.79 (0.78-0.79) |
| 2014 | 0.49 (0.49-0.5) | 0.47 (0.47-0.48) |
| 2015 | 0.52 (0.51-0.52) | 0.4 (0.4-0.41) |
|  |  |  |
| **Constant** | 2.84 (2.79-2.89) | 4.75 (4.71-4.78) |

Table S3. Standardized difference between the distribution of covariates between beneficiaries or not beneficiaries of BFP within 6 months before and after applying propensity score weights.

|  | **30-69 years** | | **0-100 years** | |
| --- | --- | --- | --- | --- |
|  | **SD** | | **SD** | |
| **Covariates** | **Prior weighting** | **After weighting** | **Prior weighting** | **After weighting** |
| **Gender** | -0.177 | -0.036 | -0.064 | -0.005 |
| **Age (at entry year)** | -0.281 | -0.001 | -0.735 | -0.096 |
| **Race/ethnicity** | 0.108 | 0.021 | 0.169 | 0.051 |
| **Education** | -0.089 | -0.036 | -0.187 | -0.064 |
| **Region** | -0.129 | -0.030 | -0.226 | -0.075 |
| **Area of residence** | 0.077 | 0.026 | 0.122 | 0.045 |
| **Household material** | 0.088 | 0.018 | 0.116 | 0.029 |
| **Sanitation** | 0.165 | 0.047 | 0.266 | 0.099 |
| **Household crowding (tercile)** | 0.535 | 0.034 | 0.588 | 0.090 |
| **Year of application** | -0.161 | -0.028 | -0.198 | -0.089 |

SD: Standardized difference

Table S4. Robustness checks using several statistical approaches to investigate the association between BFP receipt and mortality.

| **Mortality Outcomes** | **Cox model with treatment as time-varying^1^** | | **Cox model w. IPTW^2^** | | **MSM with exclusions on time to receive the benefit^3^** | | **Risk set matching^4^** | | |
| --- | --- | --- | --- | --- | --- | --- | --- | --- | --- |
|  | **Events** | **HR_adj_ (95%CI)** | **Events** | **HR_iptw_ (95%CI)** | **Events** | **HR (95%CI)** | **Events** | **HR (95%CI)** | **HR_adj_ (95%CI)** |
| **Premature mortality, all subjects 30-69 years** | **(N=4,855,324 subjects)** | | **(N=4,468,369 subjects)** | | **(N=3,668,646 subjects)** | | **(N=2,687,772 matched subjects)** | | |
| CVD overall | 15,292 | 1.04 (1.00-1.07) | 15,292 | 0.96 (0.93-1.00) | 12,675 | 1.09 (1.04-1.14) | 4,921 | 1.13 (1.06-1.19) | 1.12 (1.05-1.19) |
| Ischaemic heart diseases | 5,485 | 1.08 (1.02-1.15) | 5,485 | 1.02 (0.95-1.10) | 4,507 | 1.15 (1.07-1.23) | 1,771 | 1.24 (1.12-1.36) | 1.26 (1.14-1.40) |
| Cerebrovascular diseases | 4,151 | 1.08 (1.01-1.16) | 4,151 | 1.00 (0.92-1.09) | 3,445 | 1.09 (1.00-1.18) | 1,306 | 1.06 (0.95-1.19) | 1.02 (0.90-1.14) |
| All-cause mortality | 60,893 | 1.03 (1.02-1.05) | 60,893 | 0.98 (0.96-1.00) | 49,062 | 1.06 (1.03-1.08) | 19,420 | 1.13 (1.10-1.16) | 1.12 (1.09-1.16) |
|  |  |  |  |  |  |  |  |  |  |
| **All age groups mortality, 10M subject sample** | **(N=17,981,582 subjects)** | | **(N=16,736,067 subjects)** | | **(N=12,914,842 subjects)** | | **(N=5,705,918 matched subjects)** | | |
| CVD overall | 23,389 | 1.03 (1.00-1.05) | 23,389 | 1.03 (0.99-1.07) | 19,325 | 1.01 (0.97-1.05) | 3,283 | 1.07 (1.00-1.15) | 1.08 (1.01-1.17) |
| Ischaemic heart diseases | 7,471 | 1.10 (1.04-1.16) | 7,471 | 1.08 (1.01-1.16) | 6,196 | 1.05 (0.98-1.12) | 927 | 1.23 (1.07-1.40) | 1.27 (1.10-1.46) |
| Cerebrovascular diseases | 6,722 | 1.05 (0.99-1.11) | 6,722 | 1.05 (0.98-1.14) | 5,562 | 1.02 (0.95-1.09) | 772 | 1.03 (0.89-1.19) | 1.05 (0.90-1.23) |
| All-cause mortality | 106,807 | 0.98 (0.97-0.99) | 106,807 | 0.97 (0.95-0.99) | 82,538 | 0.92 (0.90-0.93) | 14,832 | 1.05 (1.02-1.09) | 1.05 (1.02-1.09) |

^1^Cox regression model adjusted for sex, age, race/ethnicity, education, urban/rural area of residency, household building material, sanitation, household crowding, region of residency and year of application and with cluster robust standard errors.

^2^Cox regression model weighted by IPTW considering as treated those that start receiving BFP within the first 6 months and untreated as those that did not start receiving BFP within the same time after application to CadÚnico. Specifically for this analysis, there was 16,736,067 individuals for all ages (pyr=38,087,586.6) and 4,468,369 individuals from 30 to 69 years of age (pyr=9,071,515), as individuals with <6 months of follow-up were not included in the analysis.

**^3^**Marginal Structure Models (MSM) after removing individuals that received BFP in the same day of application to the cohort.

**^4^**HR obtained using Cox regression models within the risk set matched dataset

in (i) second to last column - without additional regressors, and ^2^ last column (HR_adj_) -additionally controlling for the following covariates that were predictors used in the creation of the propensity scores: age as continuous, race, area of residency, household building material and sanitation.

Table S5. Sensitivity analysis using MSM to estimate the causal effect of BFP on cardiovascular and all-cause mortality stratified by age groups.

|  | | ***Premature mortality*** | | | |  |  | ***All age groups mortality*** | | | |
| --- | --- | --- | --- | --- | --- | --- | --- | --- | --- | --- | --- |
|  | | **CVD mortality** | | **All cause mortality** | |  |  | **CVD mortality** | | **All cause mortality** | |
|  | **N** | **Events** | **HR (95%CI)** | **Events** | **HR (95%CI)** |  | **N** | **Events** | **HR (95%CI)** | **Events** | **HR (95%CI)** |
| *Age groups* |  |  |  |  |  | *Age groups* |  |  |  |  |  |
| 30-34y | 1844935 | 800 | 0.88 (0.74-1.06) | 7776 | 0.96 (0.90-1.01) | 0-9y | 5903817 | 311 | 0.79 (0.60-1.05) | 7628 | 0.85 (0.80-0.90) |
| 35-39y | 829500 | 860 | 0.91 (0.76-1.07) | 5412 | 0.96 (0.90-1.03) | 10-19y | 1657828 | 164 | 0.83 (0.57-1.20) | 3663 | 0.82 (0.76-0.89) |
| 40-44y | 588522 | 1181 | 1.04 (0.89-1.21) | 5824 | 0.93 (0.87-1.00) | 20-29y | 1826411 | 464 | 0.86 (0.68-1.08) | 6524 | 0.98 (0.92-1.05) |
| 45-49y | 478544 | 1694 | 0.98 (0.86-1.11) | 7167 | 0.92 (0.86-0.98) | 30-39y | 1634679 | 1154 | 0.86 (0.75-0.99) | 8607 | 0.99 (0.94-1.04) |
| 50-54y | 416574 | 2544 | 0.97 (0.87-1.08) | 9163 | 0.97 (0.92-1.03) | 40-49y | 854636 | 2305 | 1.10 (1.00-1.21) | 10277 | 1.05 (1.00-1.10) |
| 55-59y | 338121 | 3160 | 0.96 (0.88-1.06) | 10521 | 0.96 (0.91-1.01) | 50-59y | 631094 | 4799 | 1.16 (1.08-1.24) | 16500 | 1.12 (1.08-1.16) |
| 60-64y | 254399 | 3589 | 0.95 (0.87-1.04) | 10779 | 0.96 (0.90-1.01) | 60-69y | 310726 | 5043 | 1.08 (1.01-1.16) | 14918 | 1.12 (1.07-1.16) |
| 65-69y | 104729 | 1464 | 0.97 (0.83-1.14) | 7776 | 0.96 (0.90-1.01) | 70-79y | 72181 | 2985 | 1.02 (0.91-1.15) | 8404 | 0.95 (0.89-1.02) |
| - |  |  |  |  |  | 80 or more | 23470 | 2100 | 0.96 (0.84-1.11) | 6017 | 0.90 (0.83-0.97) |

Table S6. Sensitivity analysis using MSM to estimate the causal effect of BFP on cardiovascular and all-cause mortality among individuals living in municipalities with lower underreporting of deaths (<0.5%).

| ***Premature mortality (N=* 3,168,629*; pyr=8,091,749.3)*** | | | | | ***All age groups mortality* *(N=* 11,081,007*; pyr=*29,543,019.5*)*** | | | | | |
| --- | --- | --- | --- | --- | --- | --- | --- | --- | --- | --- |
|  | **CVD mortality^1^** | | **All-cause mortality** | |  | **CVD mortality** | | **All-cause mortality** | | |
|  | **Events** | **HR (95%CI)** | **Events** | **HR (95%CI)** |  | **Events** | **HR (95%CI)** | **Events** |  | **HR (95%CI)** |
|  |  |  |  |  |  |  |  |  |  |  |
| Overall | 11,012 | 1.00 (0.95-1.05) | 42,082 | 0.99 (0.96-1.01) | Overall | 16,296 | 0.96 (0.84-1.09) | 70427 |  | 1.00 (0.94-1.06) |
|  |  |  |  |  |  |  |  |  |  |  |
| IBP levels^1^ |  |  |  |  | IBP levels^2^ |  |  |  |  |  |
| IBP q1 (less deprived) | 2,311 | 1.16 (1.05-1.29) | 9,079 | 1.09 (1.03-1.15) | IBP q1 (less deprived) | 3,179 | 1.35 (1.01-1.80) | 13,809 |  | 1.11 (0.97-1.27) |
| IBP q2 | 3,042 | 1.00 (0.92-1.09) | 10,574 | 1.01 (0.97-1.06) | IBP q2 | 4,482 | 0.99 (0.79-1.25) | 17,537 |  | 0.90 (0.81-1.01) |
| IBP q3 | 3,056 | 0.98 (0.89-1.08) | 11,733 | 0.99 (0.94-1.04) | IBP q3 | 4,605 | 0.87 (0.68-1.10) | 20,056 |  | 1.06 (0.95-1.20) |
| IBP q4 | 2,129 | 0.91 (0.80-1.02) | 8,519 | 0.92 (0.86-0.97) | IBP q4 | 3,311 | 0.88 (0.64-1.22) | 15,296 |  | 0.94 (0.81-1.07) |
| IBP qt5 (more deprived) | 474 | 0.89 (0.69-1.15) | 2,177 | 0.78 (0.69-0.88) | IBP qt5 (more deprived) | 719 | 0.45 (0.23-0.88) | 3,729 |  | 0.83 (0.62-1.11) |
|  |  |  |  |  |  |  |  |  |  |  |
| Family Health Strategy (FHS)^1^ |  |  |  |  | Family Health Strategy (FHS)^2^ |  |  |  |  |  |
| High coverage (≥77%) | 1,891 | 0.94 (0.85-1.03) | 7,923 | 0.91 (0.86-0.96) | High coverage (≥61%) | 2,742 | 0.74 (0.57-0.96) | 12,049 |  | 0.98 (0.88-1.10) |
| Medium coverage (42-77%) | 3,392 | 1.04 (0.95-1.14) | 13,791 | 1.01 (0.97-1.05) | Medium coverage (33-61%) | 5,268 | 1.08 (0.86-1.36) | 24,189 |  | 1.06 (0.95-1.17) |
| Lower coverage (≤42%) | 5,685 | 1.01 (0.93-1.09) | 20,319 | 1.00 (0.97-1.04) | Lower coverage (≤33%) | 8,233 | 1.09 (0.89-1.34) | 33,967 |  | 0.94 (0.85-1.04) |

^1^44 premature CVD mortality and 149 premature all-cause mortality missing values for FHS coverage.

^2^53 CVD mortality and 222 all-cause mortality missing values for FHS coverage.

# Supplementary Figures

**A.**

**B.**
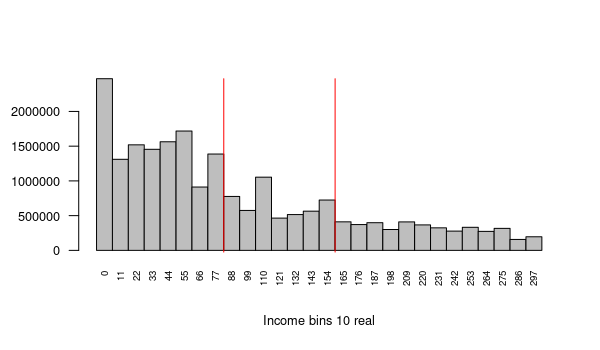


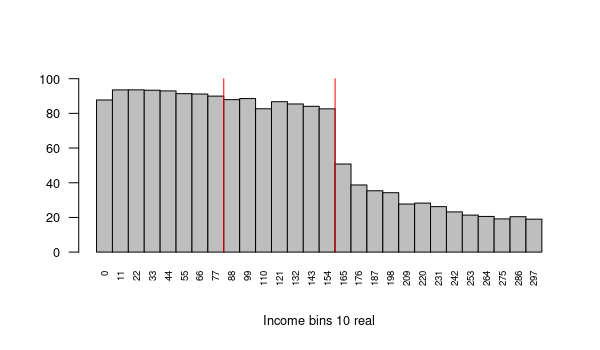


Figure S1. Distribution of standardized monthly per capita income in (A) numbers and (B) percentages.


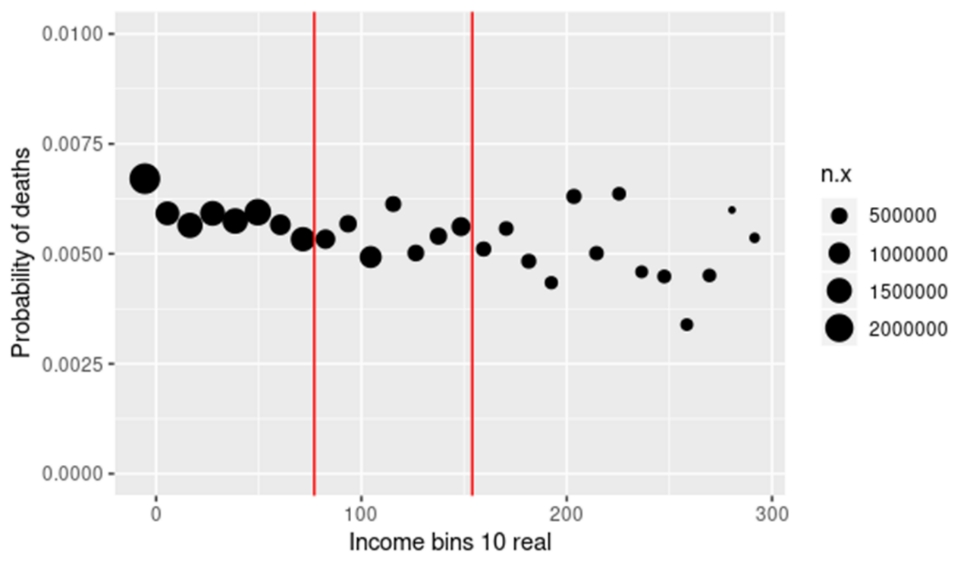


Figure S2. Distribution of probability of death over standardized monthly per capita income.


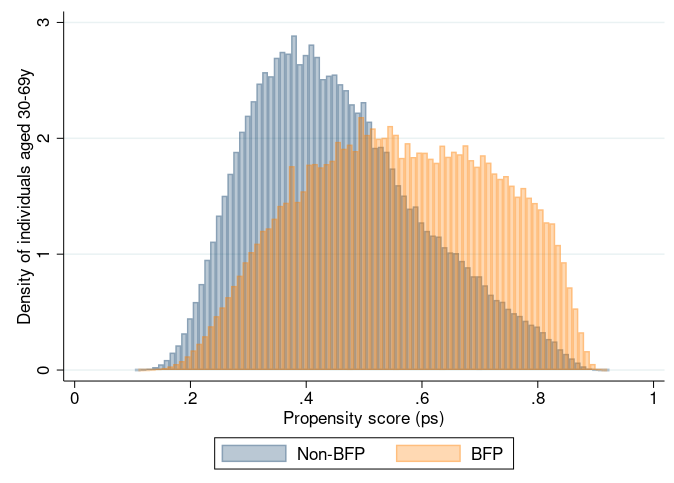
**A.**

**B.**


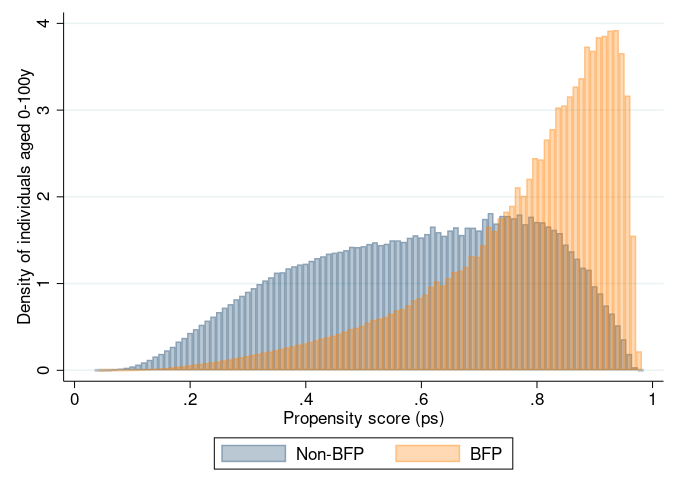


Figure S3. Propensity score distribution for the analysis 2.2 Cox regression weighting by inverse probability of treatments weights (IPTW) considering beneficiaries or not beneficiaries of BFP within 6 months after applying to the cohort among A. 30 to 69 years old, and B. all ages (0-100 years old).

References

1. Barbosa GCG, Ali MS, Araujo B, Reis S, Sena S, Ichihara MYT, Pescarini J, Fiaccone RL, Amorim LD, Pita R, Barreto ME, Smeeth L and Barreto ML. CIDACS-RL: a novel indexing search and scoring-based record linkage system for huge datasets with high accuracy and scalability. *BMC Medical Informatics and Decision Making*. 2020;20:289.

2. Pescarini JM, Craig P, Allik M, Amorim L, Ali S, Smeeth L, Barreto ML, Leyland AH, Aquino EML and Katikireddi SV. Evaluating the impact of the Bolsa Familia conditional cash transfer program on premature cardiovascular and all-cause mortality using the 100 million Brazilian cohort: a natural experiment study protocol. *BMJ Open*. 2020;10:e039658.

3. Austin PC and Stuart EA. Moving towards best practice when using inverse probability of treatment weighting (IPTW) using the propensity score to estimate causal treatment effects in observational studies. *Statistics in Medicine*. 2015;34:3661-3679.
